# Integrating single-cell and bulk transcriptomic analyses to explore key lactylation-related genes in benign prostatic hyperplasia

**DOI:** 10.1038/s41598-026-54516-x

**Published:** 2026-05-25

**Authors:** Xiaotong Pang, Deyong Zheng, Rongbin Zhou, Kun Dong, Fubo Wang

**Affiliations:** 1 Department of Urology, The First Affiliated Hospital of Guangxi Medical University, Guangxi Medical University, Guangxi, 530021 China; 2 Center for Genomic and Personalized Medicine, Guangxi Key Laboratory for Genomic and Personalized Medicine, University Engineering Research Center of Digital Medicine and Healthcare, Guangxi Medical University, Nanning, 530021 Guangxi China; 3 Department of Urology, The Second Affiliated Hospital of Fujian Medical University, Quanzhou, 362000 China; 4 School of Life Sciences, Guangxi Medical University, No. 22, Shuangyong Road, Qingxiu District, Nanning, 530021 Guangxi China; 5 Department of Organ Transplantation, The First Affiliated Hospital of Guangxi Medical University, Nanning, China

**Keywords:** BPH, Machine learning, Single-cell, Lactate, Biomarkers, Cell biology, Computational biology and bioinformatics, Diseases, Urology

## Abstract

Benign prostatic hyperplasia (BPH) is a prevalent age-related disorder characterized by chronic inflammation, metabolic dysregulation, and abnormal cellular proliferation. Protein lactylation, an emerging post-translational modification closely associated with cellular metabolism, has been implicated in the pathogenesis of various diseases. However, its role and associated molecular features in BPH remain uncharacterized. We integrated publicly available single-cell RNA sequencing (scRNA-seq) and bulk RNA-seq datasets derived from BPH and normal prostate tissues. Utilizing machine learning algorithms (LASSO, Random Forest, and Boruta) strictly as feature selection tools, we identified key lactylation-related candidate genes. Subsequently, employing the BPH-1 cell line in vitro, we preliminarily investigated the potential biological functions of the prioritized epithelial target, ANXA2, through siRNA-mediated knockdown, Western blotting, ELISA, and cell proliferation assays. We computationally identified ANXA2 and IFI27 as key lactylation-related candidate genes, both of which were significantly downregulated in BPH tissues. scRNA-seq data revealed their cell-type specificity, demonstrating that ANXA2 is predominantly expressed in epithelial cells, whereas IFI27 is primarily expressed in endothelial cells. Furthermore, we uncovered a potential link between metabolism and inflammation involving ANXA2. Specifically, ANXA2 knockdown in BPH-1 cells was associated with alterations in glycolytic gene expression and a concomitant reduction in lactate production. Our data suggest that this decreased lactate level is associated with elevated secretion of the proinflammatory cytokine IL-6 and enhanced cellular proliferation. Additionally, cellular trajectory and communication analyses indicated that ANXA2⁺ epithelial cells and IFI27⁺ endothelial cells represent distinct subpopulations characterized by enhanced intercellular signaling capacities, suggesting their potential roles as critical molecular hubs within the BPH microenvironment. This study presents the first single-cell resolution landscape of lactylation-related gene activity in BPH. We computationally identified ANXA2 and IFI27 as key lactylation-related candidate genes. Importantly, our study suggests a potential link whereby ANXA2 deficiency is associated with BPH cell proliferation and potential metabolic and inflammatory alterations. These findings provide valuable new insights into the underlying pathophysiological mechanisms of BPH and lay the groundwork for the development of targeted therapeutic strategies.

**Supplementary Information:**

The online version contains supplementary material available at 10.1038/s41598-026-54516-x.

## Introduction

BPH is a non-malignant proliferative disorder of the prostate tissue that commonly occurs in middle-aged and elderly men. It primarily develops in the transitional zone and involves the concurrent proliferation of stromal and epithelial cells, often resulting in a series of lower urinary tract symptoms^1^. Epidemiological data indicate that the prevalence of BPH increases markedly with age, affecting approximately half of men over 50 years old and exceeding 80% in those over 80 years^2^. Although BPH rarely leads directly to death, its related complications—such as acute urinary retention, urinary tract infection, and renal dysfunction—impose a substantial burden on healthcare systems and contribute significantly to the global medical and economic burden^3^. The pathophysiology of BPH remains incompletely understood but appears to be multifactorial. Androgens play a promoting role, while other factors such as altered hormonal ratios, chronic inflammation, metabolic disorders, and aberrant growth factor signaling further drive disease initiation and progression^4^. Notably, recent evidence highlights oxidative stress, inflammation, and metabolic syndrome as key contributors to the pathogenesis of BPH^5^.

In recent years, lactate has evolved from being regarded merely as the terminal product of glycolysis to being recognized as a crucial signaling molecule involved in the regulation of various physiological and pathological processes^6^. One of its important mechanisms is mediated through a novel post-translational protein modification—lysine lactylation. Accumulating evidence has demonstrated that lactylation plays a pivotal role in processes such as cell fate determination, inflammatory responses, tumorigenesis, and metabolic reprogramming^7^. In fact, clinical clues regarding the role of aberrant lactate metabolism in the pathological progression of prostate diseases have long existed. Early clinical studies detected significant abnormalities in lactate dehydrogenase (LDH) isozymes within prostatic fluid; the proportion of prostate cancer patients exhibiting an LDH-V/I ratio greater than 3 was as high as 80%, and this ratio also reached 14.4% in uninfected BPH patients. This suggests that LDH-mediated alterations in lactate metabolism serve as early-detectable metabolic markers during the pathological progression of prostate tissue. More importantly, recent studies have confirmed that lactate and lactylation modifications act as critical hubs in the crosstalk between the metabolic state and the immune-inflammatory microenvironment in prostate diseases. Previous research has demonstrated that a high-glucose environment can inhibit the glycolytic activity of prostate epithelial cells by downregulating PDK4 expression, leading to a significant decrease in intracellular lactate levels. This metabolic shift subsequently directly promotes the abnormal proliferation of BPH epithelial cells and suppresses their apoptosis; conversely, restoring lactate production can effectively inhibit BPH progression. Given that the pathophysiological hallmarks of BPH—including chronic inflammation, metabolic dysregulation, and the aberrant proliferation of stromal and epithelial cells—are closely associated with the functional roles of lactylation^8^, it is highly probable that lactylation plays a significant role in the pathogenesis and development of BPH. However, the specific target genes and molecular regulatory networks underlying lactylation in BPH remain largely unexplored.

The advent of scRNA-seq technology has greatly advanced our understanding of cellular heterogeneity in complex diseases such as BPH, enabling the identification of novel cellular subpopulations, delineation of cell states, and elucidation of potential lineage relationships at single-cell resolution^8,9^. When integrated with advanced computational approaches such as machine learning, it becomes possible to uncover complex patterns from high-dimensional scRNA-seq data, construct predictive models, and identify key biomarkers—providing powerful analytical tools for deciphering disease mechanisms^10,11^. Nonetheless, within the highly heterogeneous prostatic microenvironment, accurately identifying and functionally validating key regulatory genes in specific cell populations remains a major challenge^12^. In particular, for emerging post-translational modifications such as protein lactylation, the expression patterns, regulatory roles, and contributions of related genes to cellular heterogeneity in different BPH cell types remain poorly characterized at the single-cell level. Therefore, elucidating the functions of lactylation-related genes in BPH may provide critical insights into disease mechanisms, facilitate the discovery of cell type-specific biomarkers, and inspire the development of novel therapeutic strategies.

## Methods

### Data sources

The gene expression data for benign prostatic hyperplasia (BPH) were obtained from the Gene Expression Omnibus (GEO) database (https://www.ncbi.nlm.nih.gov/geo/), including datasets GSE132714, GSE65343, GSE104749, GSE119195, GSE7307, and GSE30994, comprising a total of 47 BPH samples and 16 healthy control samples. To identify diagnostic biomarkers and evaluate their predictive performance for BPH onset, GSE65343, GSE104749, GSE119195, GSE7307, and GSE30994 were merged into a meta-cohort termed MetaGSE, serving as the training set, which included 29 BPH samples and 12 normal controls. The dataset GSE132714, containing 18 BPH samples and 4 normal controls, was used as an external validation set. In addition, the scRNA-seq data for BPH were obtained from GSE172357, which included 5 BPH samples and 3 healthy control samples. The raw data were processed using the R package “limma”, with normalization performed via the normalizeBetweenArrays function, and log₂ transformation applied when not previously conducted. To eliminate batch effects among datasets, the ComBat function from the R package “sva” was employed, ensuring the homogeneity of the merged MetaGSE dataset. Gene lists were curated based on the intersection of unique genes across datasets, and the expression matrices were standardized for subsequent analyses. The lactylation-related gene set comprised 327 genes previously reported to be associated with the lactylation process^13^. The detailed gene list is provided in Supplementary Table S1.

### Single-cell analysis

Single-cell RNA sequencing analysis was performed using the Seurat package in R. Cells were filtered based on gene counts (200–6000 features) and mitochondrial content (< 25%). To optimize detection accuracy, we estimated the expected doublet rate for each sample to be 6.0% based on the initial cell recovery rate. Following computational classification, we retained only the high-quality cells that were reliably annotated as “singlets.” The data were normalized using the LogNormalize method, and technical covariates, including cell cycle scores and ribosomal percentage, were regressed out. Dimensionality reduction was conducted with PCA, followed by batch correction using Harmony, and visualization was performed with t-SNE based on 18 dimensions. Clustering was optimized using the Louvain algorithm across multiple resolutions. Cell-type annotation combined automated classification via SingleR with manual curation based on differentially expressed genes, identifying epithelial cells, endothelial cells, T cells, fibroblasts, macrophages, smooth muscle cells, B cells, and mast cells.

### Assessment of lactylation gene set activity based on multiple scoring algorithms

To evaluate the transcriptional activity of lactylation-related genes in the single-cell transcriptome, multiple gene set scoring algorithms were employed for integrated analysis. First, the AUCell algorithm was used to assess the activity of the lactylation-related gene set in each cell by calculating the area under the cumulative distribution curve (AUC) of gene expression rankings. This involved ranking genes for each cell and computing AUC scores for the gene set. In parallel, the irGSEA package was applied to compute UCell and singscore metrics via the irGSEA.score function. UCell quantifies gene set activity based on normalized single-cell expression ranks, while singscore calculates the mean rank score of genes within the lactylation-related gene set^14^. Additionally, the ssGSEA method from the GSVA package was implemented, and Seurat’s AddModuleScore function was used to compute the weighted average expression of the lactylation gene set per cell^15^. To integrate results from different algorithms, all scores were standardized using z-score scaling and 0–1 normalization, after which the mean value of the five algorithm-derived scores was calculated as the composite activity score. Finally, all scoring results were added to the meta. data slot of the Seurat object for subsequent analyses.

### Enrichment analysis

The clusterProfiler package was used to perform Gene Ontology (GO) and Kyoto Encyclopedia of Genes and Genomes (KEGG) pathway enrichment analyses to evaluate the functional characteristics of lactylation-related genes^16^. First, gene symbols were converted to Entrez Gene IDs using the org.Hs.eg.db database, and unmatched genes were filtered out. Subsequently, GO enrichment analysis was conducted using the enrichGO function with the ontology set to “ALL” and multiple testing correction performed using the Benjamini-Hochberg (BH) method. For KEGG pathway analysis, the enrichKEGG function was applied, with similar p-value cutoffs used for significance determination.

### Feature gene selection via machine learning algorithms

Multiple machine learning algorithms were employed to identify optimal feature genes, including Least Absolute Shrinkage and Selection Operator (LASSO) regression, Random Forest (RF), and the Boruta algorithm^17^. First, LASSO regression was applied for feature selection. By introducing a regularization term, this method effectively enhances predictive accuracy while reducing the risk of overfitting. The LASSO model was optimized through 10-fold cross-validation, and model performance was further assessed by examining the LASSO coefficient and cross-validation plots. In parallel, a Random Forest ensemble model consisting of 500 decision trees was constructed. The optimal number of trees was determined by analyzing the error rate curve, and the importance of each feature was evaluated based on the mean decrease in Gini impurity. In addition, the Boruta algorithm was implemented for feature selection by comparing the importance of real features with that of randomly generated “shadow features,” thereby enabling the robust identification of all relevant variables. By integrating the results from these algorithms, we ultimately identified genes that had significant effects on disease progression, and the optimal feature genes were determined by the overlap among the results of the LASSO, Random Forest, and Boruta analyses.

### Cell-cell communication analysis

Cell-cell communication analysis was performed using CellChat to infer intercellular signaling networks from single-cell transcriptomic data^18^. The analytical workflow followed established protocols, utilizing the human CellChatDB as the reference database for ligand-receptor interactions. After constructing the CellChat object, overexpressed genes and interactions among the nine identified cell populations were determined. Communication probabilities were calculated using the triMean method, and interactions were filtered to include only those involving cell groups with at least 10 cells. The resulting communication networks were then aggregated, and the interaction strength between different cell types was quantified. Specific signaling pathways of interest were examined in detail. The inferred intercellular communication networks were visualized using bubble plots, chord diagrams, and heatmaps, allowing the identification of dominant ligand-receptor pairs and their relative contributions to overall cell communication.

### Trajectory analysis

To reconstruct cellular developmental trajectories from single-cell RNA sequencing data, Monocle was employed for pseudotime analysis^19^. The RNA expression count matrix was first extracted from the Seurat object, and AnnotatedDataFrame objects containing gene annotations and sample metadata were created to initialize the CellDataSet. Subsequently, normalization and dispersion estimation were performed using the estimateSizeFactors and estimateDispersions functions to ensure analytical robustness. Genes with expression levels greater than 1.0 and expressed in at least 50 cells were selected as candidate genes. Differential expression analysis based on seurat_clusters was then conducted to identify key genes for trajectory ordering. Dimensionality reduction was achieved using the DDRTree method, and cellular pseudotime trajectories were inferred with the orderCells function. Finally, trajectories were visualized using the plot_cell_trajectory function.

### Immunohistochemistry

This study utilized six pairs of matched benign prostatic hyperplasia and adjacent normal prostate tissue samples obtained from the Second People’s Hospital of Nanning. All study procedures were conducted in accordance with the principles of the Declaration of Helsinki, and written informed consent was obtained from all participants prior to the commencement of the study. The study protocol was approved by the Medical Ethics Committee of the Second People’s Hospital of Nanning (Ethics Code: Y2025111). Paraffin-embedded tissue sections were deparaffinized, rehydrated, and subjected to heat-induced antigen retrieval in citrate buffer. Endogenous peroxidase activity was blocked with 3% H₂O₂ for 25 min, followed by 3% bovine serum albumin (BSA) blocking for 30 min to prevent nonspecific binding. The sections were then incubated overnight at 4 °C with primary antibodies against ANXA2 (CLOUD-CLONE, PAB944Hu01, 1:200) or IFI27 (Elabscience, E-AB-19105, 1:100), followed by incubation with HRP-conjugated secondary antibodies at room temperature for 50 min. Staining was developed using DAB substrate, counterstained with hematoxylin, dehydrated, and mounted. The staining intensity was semi-quantitatively evaluated using the H-score system: H-Score = Σ (pi × i) = (% of weakly stained cells × 1) + (% of moderately stained cells × 2) + (% of strongly stained cells × 3), where i represents the staining intensity (0–3), and pi indicates the percentage of positively stained cells at each intensity level. The total score ranges from 0 to 300.

### Cell culture

The human benign prostatic hyperplasia cell line, BPH-1 (RRID: CVCL_1090), was purchased from Servicebio (Wuhan, China; STCC11201P) in March 2025. This cell line is derived from human (male) prostate tissue. The supplier provided an STR authentication report (dated Aug 09, 2022), which confirmed the identity of the cell line using STR profiling (19 loci). The analysis showed a 92.86% match to the BPH-1 profile in the Cosmic-CLP database, confirming the cell line was not misidentified. The cell line was confirmed to be free of mycoplasma contamination by routine testing. The BPH-1 cells were cultured in RPMI-1640 medium (GZ11201) supplemented with 10% fetal bovine serum (FBS) and 1% penicillin-streptomycin (P/S) under standard conditions of 37 °C and 5% CO₂ in a humidified incubator. Cells were passaged at a 1:6 ratio after digestion with 0.25% trypsin-EDTA solution for 1 min at 37 °C. The culture medium was replaced two to three times per week to maintain optimal growth conditions.

### Exogenous lactate treatment

BPH-1 cells were cultured in a medium supplemented with 10 mM or 20 mM exogenous lactate (MCE, HY-B2227) for 48 h to investigate the direct regulatory effects of lactate on IL-6 and IL-8 secretion. Upon completion of the treatment, cell culture supernatants were collected, and cytokine concentrations were quantified using ELISA.

### siRNA transfection

To knock down ANXA2 expression in BPH-1 cells, specific siRNA sequences were designed and synthesized (si-ANXA2-1: sense 5’-GGGUCUGUCAAAGCCUAUATT-3’, antisense 5’-UAUAGGCUUUGACAGACCCTT-3’; si-ANXA2-2: sense 5’-GAGUCUACAAGGAAAUGUATT-3’, antisense 5’-UACAUUUCCUUGUAGACUCTT-3’), along with a non-targeting negative control siRNA (si-NC: sense 5’-UUCUCCGAACGUGUCACGUTT-3’, antisense 5’-ACGUGACACGUUCGGAGAATT-3’) (HyCyte, HX-H-S3-07302). Transfection was performed using a non-liposomal transfection reagent (Seven Biotech, SC212) according to the manufacturer’s protocol, with 30 pmol of siRNA applied per well. After 48 h of incubation at 37 °C, Western blot analysis was conducted to evaluate transfection efficiency and identify the most effective siRNA sequence for subsequent experiments.

### Western blot analysis

Total protein was extracted from BPH-1 cells using RIPA lysis buffer supplemented with protease inhibitors, followed by centrifugation at 12,000 rpm for 10 min at 4 °C. Protein samples were denatured by boiling in 5× reducing loading buffer for 15 min, separated by SDS-PAGE, and transferred onto PVDF membranes at 350 mA for 30 min. Due to the distinct differences in molecular weights between the target proteins and their corresponding loading controls, intact membranes were co-incubated overnight at 4 °C with specific mixtures of primary antibodies. Specifically, for the detection of ANXA2, membranes were co-incubated with primary antibodies against ANXA2 (Servicebio, GB111259-100, 1:1500) and the loading control α-tubulin (Servicebio, GB15201-100, 1:1000). For the detection of Hexokinase II, a separate set of membranes was co-incubated with primary antibodies against Hexokinase II (Servicebio, GB121063, 1:5000) and the loading control ACTIN (Servicebio, GB15001, 1:3000). After washing with TBST, membranes were incubated with HRP-conjugated secondary antibodies (1:5000) at room temperature for 30 min. Chemiluminescent signals for both the target proteins and their respective loading controls were detected simultaneously on the same blots using a 1:1 mixture of ECL substrates and visualized on an SCG-W3000 Chemiluminescence Imaging System.

### Measurement of endogenous lactate production

BPH-1 cells were transfected with either siRNA-NC or siRNA-ANXA2-2. After a 48-hour incubation at 37 °C, the cell culture supernatants were collected. Endogenous lactate concentrations were quantified using a Lactate Assay Kit (Servicebio, G4308). In accordance with the manufacturer’s instructions, 5 µL of each sample was mixed with 200 µL of assay buffer and incubated at 37 °C for 5 min. Subsequently, 40 µL of enzyme solution was added, followed by an additional 5-minute incubation. The absorbance was measured at 546 nm, and the lactate concentration was calculated using the following formula: Lactate (mmol/L) = [(Sample Absorbance − Blank Absorbance)/(Standard Absorbance − Blank Absorbance)] × Standard Concentration × Dilution Factor. Concurrently, a portion of the supernatants from these transfected cells was collected for subsequent quantification of IL-6 and IL-8 via ELISA.

### Enzyme-linked immunosorbent assay (ELISA)

Cell culture supernatants were collected, diluted 1:2, and the concentrations of IL-6 and IL-8 were measured using commercial ELISA kits (Thermo Fisher, 88–7066 and 88–8086) in accordance with the manufacturer’s instructions. Briefly, 100 µL of diluted sample was added to pre-coated 96-well plates and incubated at 37 °C for 1 h. Sequential incubation with biotinylated antibodies and HRP-conjugated secondary antibodies was performed, followed by color development with TMB substrate solution for 30 min. The reaction was stopped using 2 M sulfuric acid, and absorbance was measured at 450 nm using a microplate reader. Cytokine concentrations were calculated based on a standard curve generated from recombinant proteins.

### Cell proliferation assay

Cell proliferation was assessed using the Cell Counting Kit-8 (CCK-8). BPH-1 cells were seeded into 96-well plates at a density of 5 × 10³ cells per well in 100 µL of complete culture medium and incubated at 37 °C with 5% CO₂. At designated time points (0, 24, 48, and 72 h), 10 µL of CCK-8 reagent (Servicebio, G4103) was added to each well, and the plates were incubated for an additional 2 h at 37 °C. Absorbance was measured at 450 nm using a microplate reader.

### Statistical analysis

Statistical analyses were performed using R software (version 4.2.2) and GraphPad Prism (version 10.1.2). Comparisons between groups were conducted using Student’s t-test or one-way ANOVA, as appropriate. Statistical significance was denoted as: *p < 0.05, **p < 0.01, ***p < 0.001, and ****p < 0.0001; ns indicated no significance (*p* ≥ 0.05).

## Results

### Quality control and clustering of scRNA-seq data

**Fig. 1:**
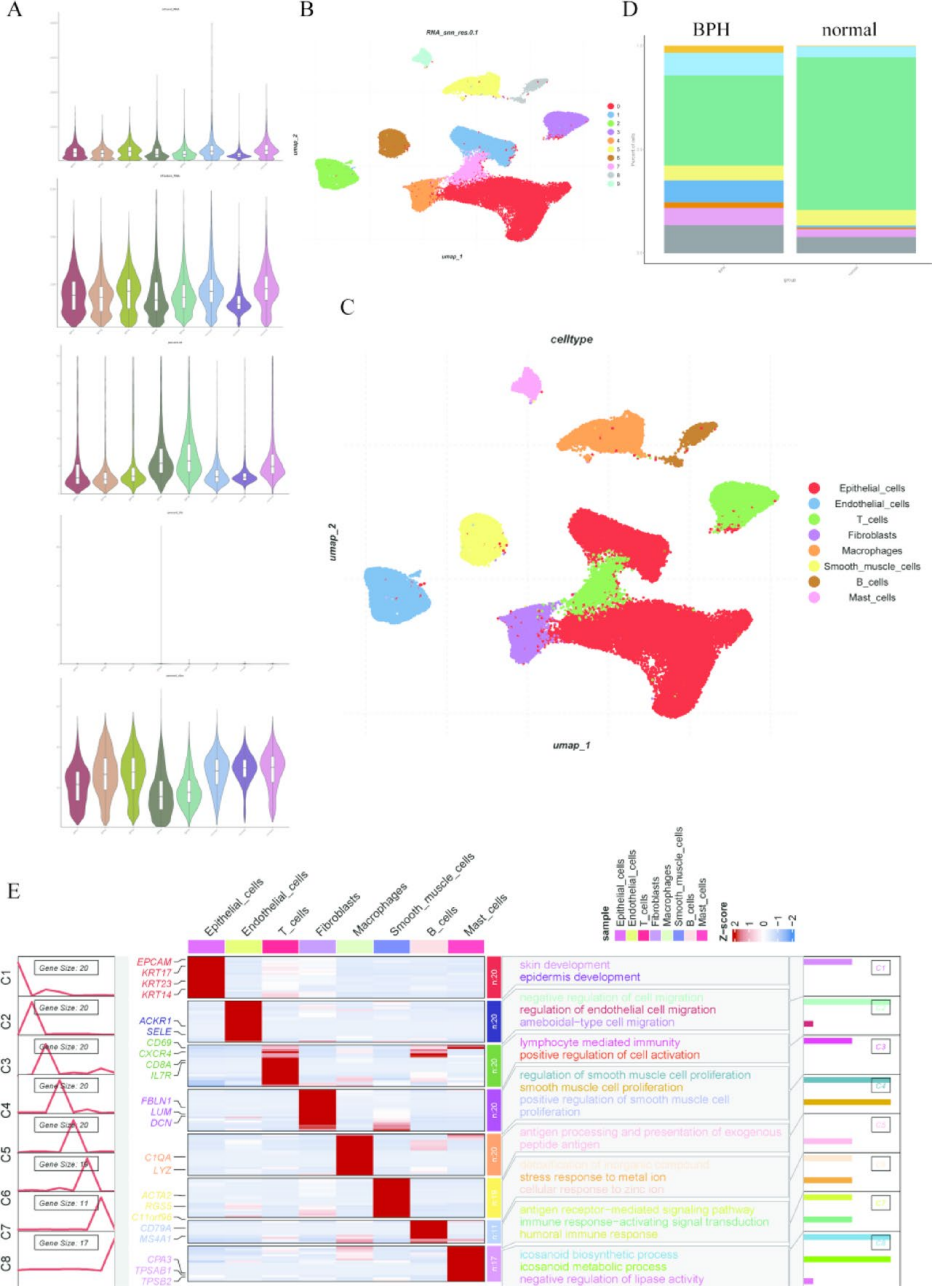
Quality control, clustering, and cell type identification of single-cell RNA sequencing data. **(A)** Quality control metrics, including RNA counts, gene numbers, and proportions of mitochondrial, hemoglobin, and ribosomal genes. **(B)** t-SNE clustering plot showing the distribution of cells across ten distinct clusters. **(C)** Identification of major cell types, including epithelial cells, endothelial cells, T cells, fibroblasts, macrophages, smooth muscle cells, B cells, and mast cells. **(D)** Comparison of the proportional distribution of each cell type between BPH and normal samples. **(E)** Expression of cell-type-specific marker genes and corresponding functional enrichment analysis.

To ensure the scientific rigor and reliability of downstream analyses, a stringent quality control pipeline was applied to the scRNA-seq dataset (Fig. 1A). During cell filtering, cells with abnormal proportions of mitochondrial, ribosomal, or hemoglobin gene expression, as well as those with low RNA sequencing depth or atypical numbers of detected genes, were excluded to minimize technical noise and remove low-quality cells. After rigorous filtering, a total of 60,396 high-quality cells were retained for subsequent analyses. To eliminate technical variation, batch effect correction was performed across all single-cell RNA-seq datasets (Supplementary Figure S1A). The t-SNE visualization demonstrated an even distribution of BPH and normal samples in the reduced dimensional space, confirming the successful removal of batch effects. Using the Seurat analysis workflow, unsupervised clustering was performed on the QC-passed cells, resulting in the identification of 10 distinct cell clusters (Fig. 1B). Based on the expression patterns of characteristic marker genes, we annotated these clusters into eight major cell phenotypes: epithelial cells, endothelial cells, fibroblasts, smooth muscle cells, T cells, B cells, macrophages, and mast cells (Fig. 1C). Comparative analysis of cell type proportions between the BPH and normal groups (Fig. 1D) revealed increased proportions of smooth muscle cells, endothelial cells, and macrophages in the BPH group, accompanied by a reduced proportion of epithelial cells. This finding underscores the critical role of stromal cell proliferation in the pathological progression of BPH. A heatmap of cell type-specific marker gene expression (Fig. 1E) clearly delineated the molecular characteristics of the eight identified cell types. Corresponding GO enrichment analyses highlighted key biological processes associated with each cell type, including skin development, regulation of endothelial cell migration, immune response, and smooth muscle cell proliferation. Moreover, UMAP density plots demonstrated the specific spatial distribution of marker genes such as S100A2, SELE, IL7R, FBLN1, IL1B, MT1A, IGKC, and TPSB2 across corresponding cell populations, further validating the accuracy of cell type annotation (Supplementary Figure S1B).

### Analysis of lactylation gene expression activity across different cell types

**Fig. 2:**
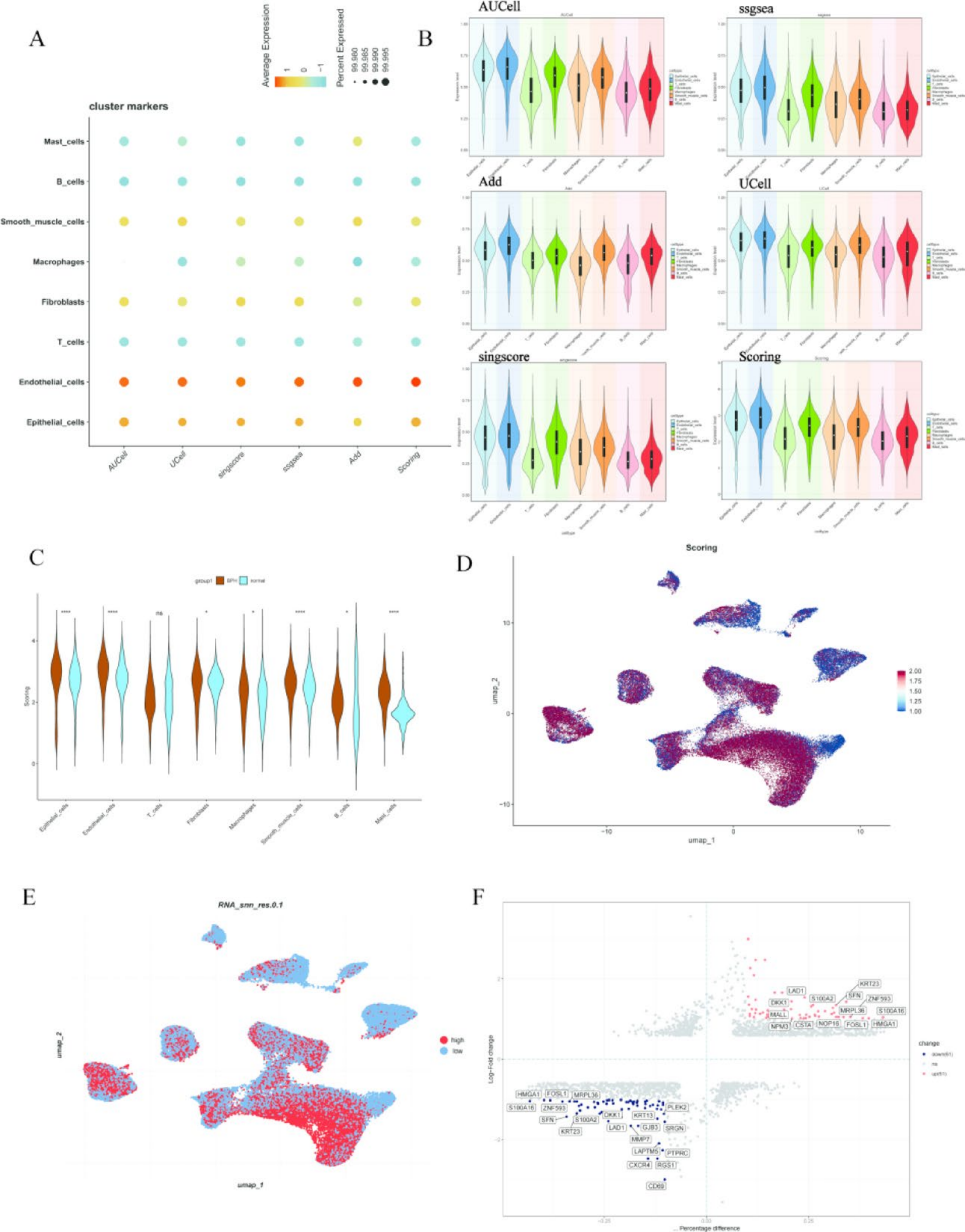
Expression scoring of lactylation-related genes across prostate cell types. **(A)** Bubble plot showing lactylation expression scores among different cell types, where color intensity and bubble size indicate expression strength. **(B)** Violin plots displaying lactylation gene activity evaluated by six algorithms, showing the highest activity in endothelial and epithelial cells. **(C)** Comparison of lactylation gene expression activity between BPH and normal tissues. **(D)** t-SNE plot illustrating the spatial distribution of lactylation expression activity. **(E)** Classification of cells into high-score (red) and low-score (blue) groups based on the median lactylation score. **(F)** Volcano plot of differentially expressed genes showing expression changes of lactylation-related genes between high- and low-scoring groups.

To evaluate the expression activity of lactylation-related genes among various cell types, we applied multiple gene set scoring algorithms—including AUCell, UCell, singscore, ssGSEA, and AddModuleScore—and calculated the mean score for each cell. The results were visualized using bubble plots and violin plots, where differences in color and size reflected variations in expression intensity (Figs. 2A-B). The bubble plot revealed that epithelial cells and endothelial cells exhibited the highest lactylation gene activity, a finding consistently observed across all scoring methods. Smooth muscle cells and fibroblasts displayed moderate activity levels, whereas immune cell populations (T cells, B cells, and macrophages) showed relatively lower activity. Although absolute scores varied slightly across algorithms, the relative ranking of cell types remained largely consistent. Comparative analysis between the BPH and normal groups demonstrated significantly higher lactylation gene activity in multiple cell types of the BPH group (Fig. 2C). Specifically, lactylation scores were markedly elevated in epithelial cells, endothelial cells, B cells, fibroblasts, smooth muscle cells, mast cells, and macrophages, whereas T cells showed no significant difference. UMAP visualization further elucidates the spatial distribution of lactylation-related gene expression signatures at the single-cell level (Fig. 2D). Cells were categorized into high-score (red) and low-score (blue) groups based on the median lactylation-related gene expression score (Fig. 2E). Differential gene expression analysis identified 250 significantly differentially expressed genes (DEGs) associated with elevated lactylation-related gene expression (Fig. 2F, Supplementary Table S2). Notably, cells with high expression of lactylation-related genes were predominantly enriched within the epithelial and endothelial cell populations. These findings suggest that lactylation-related genes may potentially participate in the pathophysiological processes of BPH, exhibiting a high degree of expression correlation particularly within epithelial and endothelial cells.

### Identification of co-expression networks associated with lactylation-related genes using hdWGCNA

**Fig. 3:**
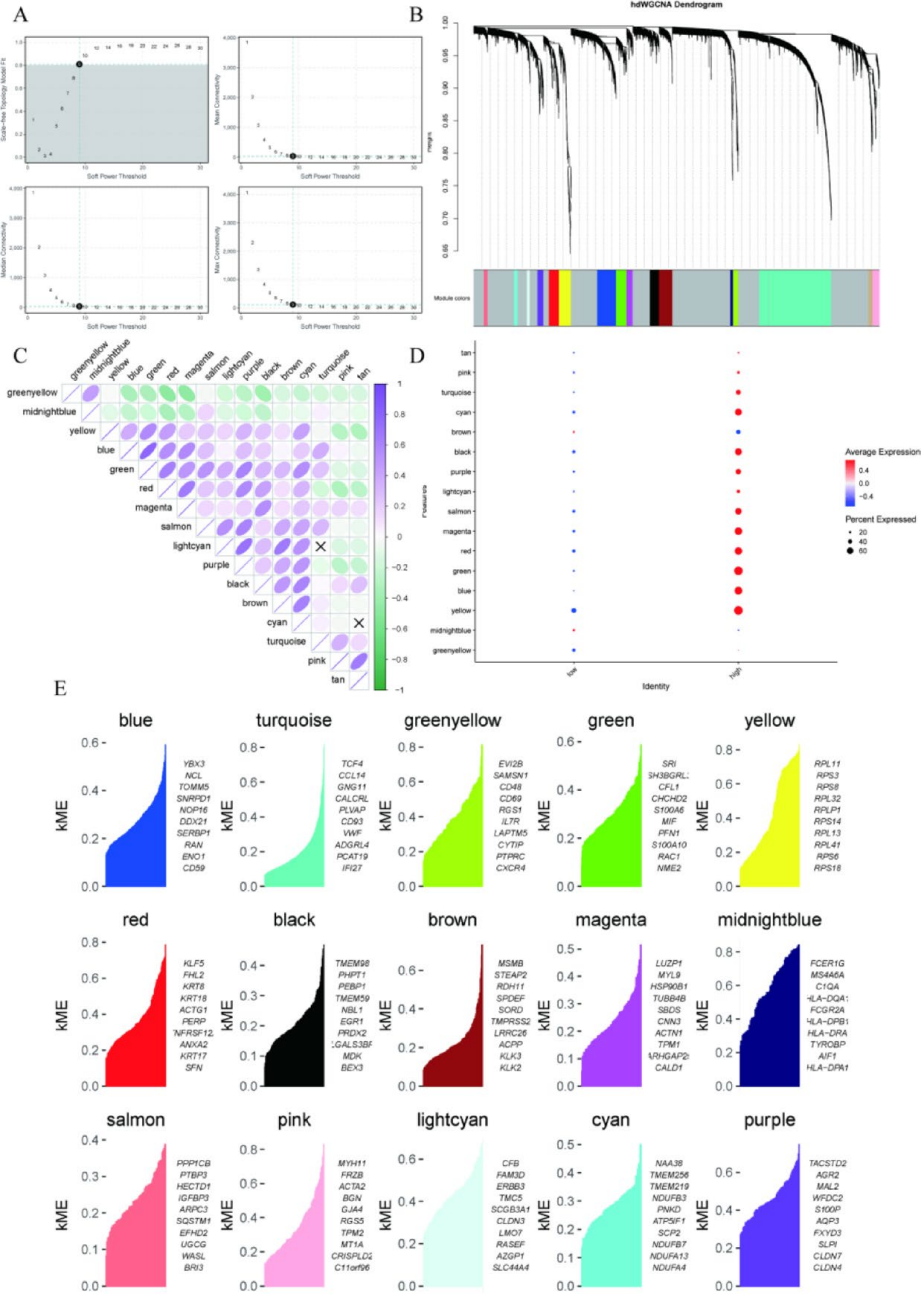
Identification of co-expression gene networks associated with lactylation using hdWGCNA. **(A)** Topological analysis of the scale-free network constructed with a soft-thresholding power of 9. **(B)** Hierarchical clustering dendrogram dividing highly variable genes into 16 distinct color-labeled modules. **(C)** Correlation matrix of module eigengenes, with color intensity indicating the strength of correlation. **(D)** Distribution of module activity between high and low lactylation expression groups. **(E)** Visualization of hub genes within key modules ranked by module membership.

Through high-dimensional weighted gene co-expression network analysis (hdWGCNA), we identified gene modules most closely associated with the high lactylation score group. First, a scale-free co-expression network was constructed using a soft threshold power of 9 (Fig. 3A), which successfully classified highly variable genes into 16 distinct modules (Fig. 3B). Figure 3C illustrates the correlation matrix among module eigengenes, providing a clear visualization of the interrelationships between modules. We further evaluated the module activity in the high lactylation score group (Fig. 3D) and found that the yellow, blue, green, red, magenta, salmon, black, cyan, turquoise, and brown modules were significantly correlated with high lactylation scores, suggesting that these modules may play key roles in the regulation of lactylation processes. Additionally, we conducted a core gene analysis within each module to identify hub genes potentially driving module-specific functions (Fig. 3E).

### Identification and functional analysis of key lactylation-related genes in BPH

**Fig. 4:**
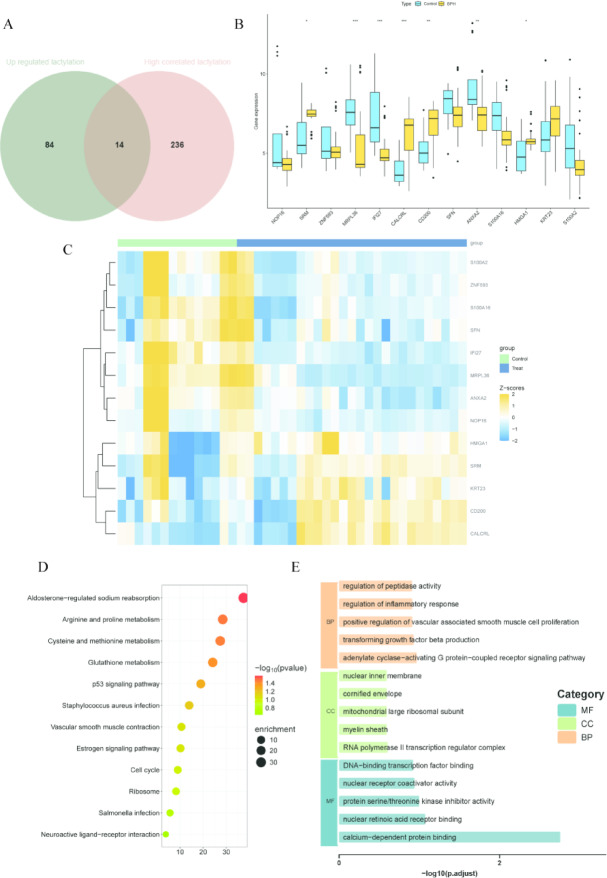
Identification and functional analysis of key lactylation-related genes. **(A)** Venn diagram showing the overlap between upregulated lactylation-related genes and highly correlated lactylation genes. **(B)** Boxplots displaying the expression differences of overlapping genes between control and BPH groups. **(C)** Heatmap illustrating the expression profiles of core genes across all samples. **(D)** KEGG pathway enrichment analysis of the overlapping genes. KEGG pathway maps are reproduced with permission from Kanehisa Laboratories^20–22^. **(E)** GO functional enrichment analysis highlighting the main biological processes associated with these genes.

First, we employed the “sva” package to correct for batch effects and normalize the raw data. Following batch correction, principal component analysis (PCA) results demonstrated a harmonious integration of samples across the various datasets (Supplementary Figures S2A-B). In the bulk RNA-seq data, we performed an integrated analysis of differentially expressed genes and lactylation-related genes with high correlation. Venn diagram analysis revealed 14 overlapping genes (Fig. 4A, Supplementary Table S3), which may play pivotal roles in the lactylation regulatory network. To verify the reliability of these candidate genes, we further examined their expression differences between non-BPH and BPH groups in the bulk RNA-seq datasets (Fig. 4B). The boxplot results showed that several genes, including SRM, CALCRL, and HMGA1, were significantly upregulated in the BPH group, while MRPL36, ANXA2, and IFI27 were markedly downregulated. The heatmap analysis (Fig. 4C) intuitively displayed the expression profiles of these 14 overlapping genes across different samples, further confirming their distinct expression patterns between BPH and control groups. To elucidate the functional significance of these key genes, we conducted KEGG pathway enrichment analysis (Fig. 4D). The results indicated that these genes were mainly involved in critical biological processes such as amino acid metabolism and the p53 signaling pathway. Additionally, GO enrichment analysis (Fig. 4E, Supplementary Table S4) revealed that these genes were primarily associated with regulation of peptide activity, positive regulation of vascular smooth muscle cell proliferation, and cell signaling pathways, suggesting their participation in essential cellular processes related to the pathogenesis of BPH.

### Identification of key lactylation-related biomarkers

**Fig. 5:**
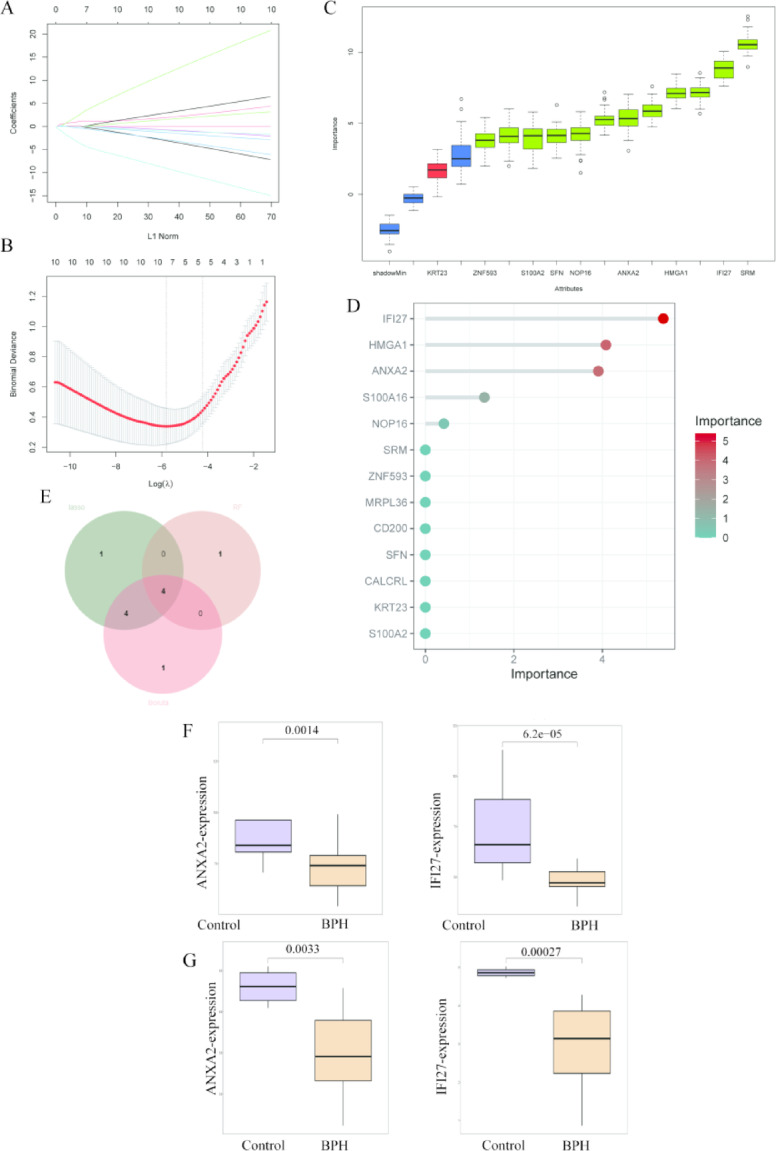
Machine learning-based identification and validation of key lactylation marker genes. **(A-B)** Feature selection using the LASSO regression model. **(C)** Distribution of gene importance scores assessed by the Boruta algorithm. **(D)** Ranking of gene importance determined by the Random Forest algorithm. **(E)** Venn diagram showing four core marker genes jointly identified by all three algorithms. **(F)** Boxplots of ANXA2 and IFI27 expression levels in the training cohort. **(G)** Differential expression analysis of candidate genes in the validation cohort.

To further screen for core marker genes with predictive value among lactylation-related genes, we employed multiple machine learning algorithms for feature selection in the training cohort. First, we applied the LASSO regression model for variable selection. Figure 5A illustrates the changes in coefficients under different L1 regularization parameters, while Fig. 5B shows the variation in binomial deviance during cross-validation as a function of log(λ). The optimal model was determined based on the minimum binomial deviance criterion. Subsequently, we used the Boruta algorithm to evaluate feature importance, with Fig. 5C displaying the distribution of importance scores for each gene. The random forest model further validated the significance of these features through importance scoring (Fig. 5D). By comparing the gene intersections identified by the LASSO, random forest, and Boruta algorithms using a Venn diagram (Fig. 5E), we identified four core marker genes jointly recognized by all three methods: IFI27, NOP16, HMGA1, and ANXA2. In the training cohort, the expression level of HMGA1 was significantly higher in the BPH group compared to controls, whereas NOP16 did not show a statistically significant difference (Supplementary Figure S3A-B). However, in the validation cohort, NOP16 remained non-significant, and HMGA1 exhibited an expression trend opposite to that observed in the training cohort (Supplementary Figure S3C-D). We then focused on the two more prominent candidates, ANXA2 and IFI27, for further analysis. In the training cohort, both ANXA2 and IFI27 were significantly downregulated in the BPH group relative to controls (Fig. 5F). In the independent validation cohort, the expression patterns of these genes were consistent with those in the training cohort (Fig. 5G).

### Single-cell validation of optimal lactylation-related diagnostic biomarkers

**Fig. 6:**
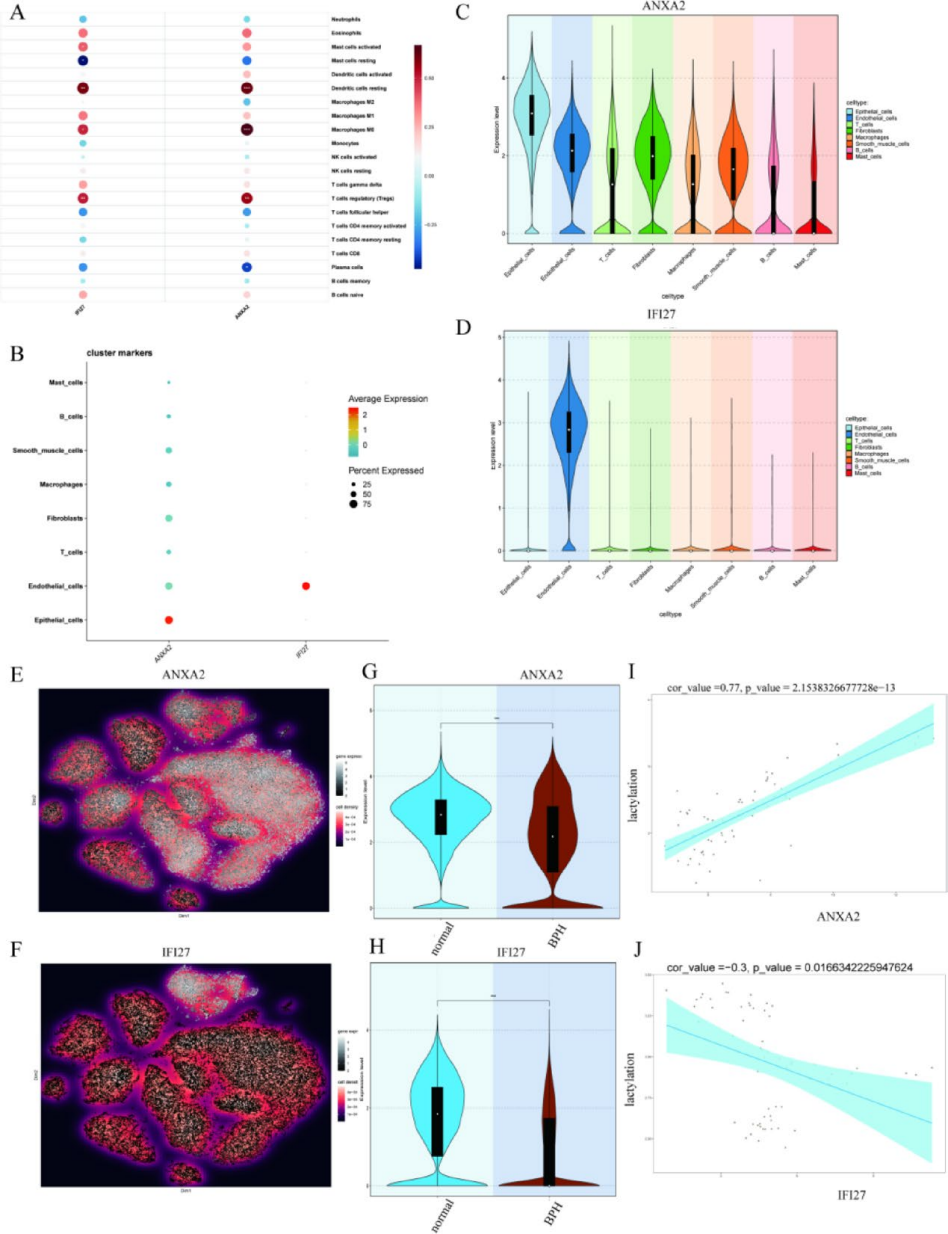
Single-cell expression characteristics of ANXA2 and IFI27 and their association with lactylation. **(A)** CIBERSORT analysis showing the correlations of the two genes with various immune cell populations. **(B)** Bubble plot illustrating the enrichment of ANXA2 in epithelial cells and IFI27 in endothelial cells. **(C-D)** Violin plots displaying the expression distributions of both genes across different cell types. **(E-F)** t-SNE plots showing the spatial expression patterns of the two genes. **(G-H)** Comparison of ANXA2 and IFI27 expression levels between BPH and normal tissues. **(I-J)** Scatter plots revealing the negative correlations between gene expression and lactylation activity.

To validate the optimal feature genes at the single-cell level, several analyses were conducted. In the bulk RNA-seq dataset, CIBERSORT analysis revealed that both genes exhibited specific correlations with multiple immune cell populations in BPH (Fig. 6A). Notably, ANXA2 showed a positive correlation with M0 macrophages and dendritic cells, whereas IFI27 was closely associated with mast cells and regulatory T cells. Cell type-specific expression analysis confirmed that ANXA2 was predominantly expressed in epithelial cells, while IFI27 was enriched in endothelial cells (Fig. 6B-D). The t-SNE spatial distribution further corroborated these expression patterns (Fig. 6E-F). When comparing BPH and normal tissues, both genes were found to be significantly downregulated in the BPH group (Fig. 6G-H), consistent with the results from the bulk RNA-seq analysis. Importantly, correlation analysis revealed that ANXA2 was positively correlated with the lactylation-related gene expression score, whereas IFI27 exhibited a negative correlation (Fig. 6I-J). These findings suggest that both genes may contribute to the pathogenesis and progression of BPH, potentially through involvement in lactate accumulation and immune cell infiltration.

.

### Cell trajectory and communication analyses reveal the roles of ANXA2⁺Epithelial cells and IFI27⁺Endothelial cells in BPH

**Fig. 7:**
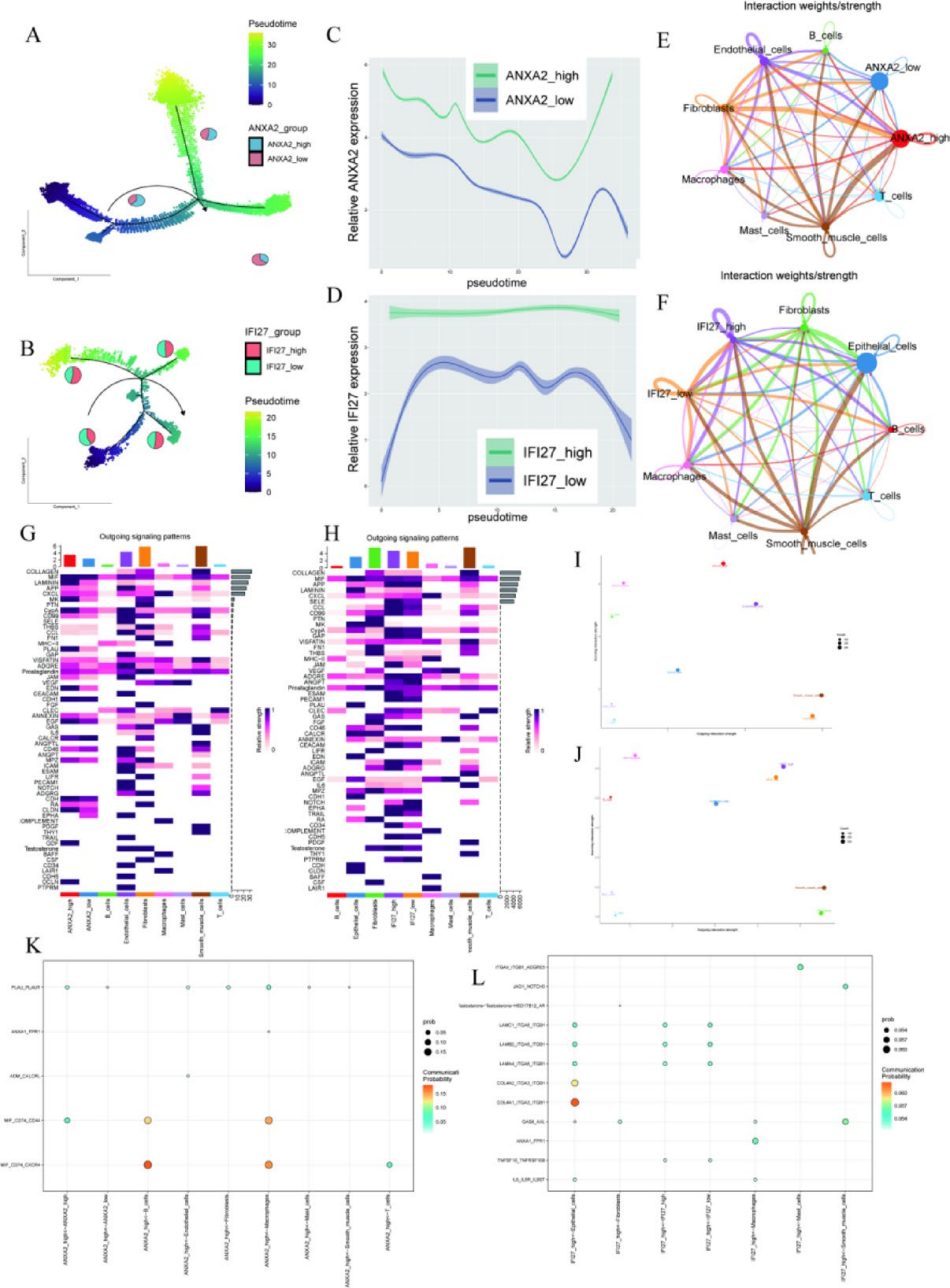
Differentiation trajectories and cell-cell communication analysis of ANXA2⁺epithelial cells and IFI27⁺endothelial cells. **(A-B)** Pseudotime trajectory analysis using Monocle2 showing the synchronized differentiation dynamics of ANXA2⁺and IFI27⁺cell subpopulations. **(C-D)** Dynamic expression changes of the two key genes along the pseudotime axis. **(E-F)** Cell-cell communication networks depicting the interaction strength between high-expression subgroups and other cell types. **(G-H)** Heatmaps illustrating the pathway enrichment patterns specific to each cell subpopulation. **(I-J)** Scatter plots of outgoing and incoming interaction strengths highlighting the enhanced signaling activity of high-expression groups. **(K-L)** Ligand-receptor bubble plots presenting detailed key communication pairs between ANXA2⁺epithelial cells and IFI27⁺endothelial cells.

Eepithelial cells were classified into ANXA2⁺and ANXA2⁻groups, while endothelial cells were divided into IFI27⁺and IFI27⁻groups. Monocle2 pseudotime analysis (Figs. 7A-B) revealed that these two highly expressed subpopulations exhibited synchronous dynamic changes during differentiation. As pseudotime progressed, the proportions of ANXA2⁺epithelial cells and IFI27⁺endothelial cells within their respective lineages gradually increased, accompanied by a progressive decline in ANXA2 expression along the pseudotime trajectory (Figs. 7C-D). These findings suggest that ANXA2⁺epithelial cells and IFI27⁺endothelial cells may represent more mature subpopulations within their respective cell lineages. Compared with ANXA2⁻cells, ANXA2⁺epithelial cells displayed significantly enhanced interaction numbers and strengths with multiple other cell types, including fibroblasts and macrophages (Fig. 7E). Similarly, IFI27⁺endothelial cells exhibited stronger intercellular communication activity than their IFI27⁻counterparts (Fig. 7F). Signaling pathway analysis revealed key communication networks involving these subpopulations. ANXA2⁺epithelial cell-associated communications were mainly enriched in MIF, EGF, and LAMININ signaling pathways (Fig. 7G, Supplementary Table S5), whereas IFI27⁺endothelial cells were predominantly engaged in CD34, Testosterone, and CCL signaling (Fig. 7H, Supplementary Table S6). Based on these bioinformatic inferences, These findings suggest that in the pathological microenvironment of BPH, ANXA2⁺epithelial cells may promote inflammatory responses, cell proliferation, and extracellular matrix remodeling, while IFI27⁺endothelial cells may function by responding to androgen signaling, regulating immune cell recruitment, and possibly contributing to angiogenesis or cell adhesion processes, collectively driving disease progression. Further analysis of overall communication strength confirmed that both ANXA2⁺epithelial cells and IFI27⁺endothelial cells exhibited higher total input and output signal intensities (Figs. 7I-J). Finally, ligand-receptor interaction analysis detailed how ligands from different cell types interacted with receptors on ANXA2⁺epithelial cells (Fig. 7K) and IFI27⁺endothelial cells (Fig. 7L). Notably, macrophages interact with ANXA2⁺ epithelial cells via the CD74-CD44/MIF axis, while epithelial-derived COL4A1 binds to the ITGA3/ITGB1 integrins on IFI27⁺ endothelial cells, potentially participating in crucial stroma-cell communication within the BPH microenvironment.

### ANXA2 regulates metabolic-inflammatory interactions in BPH cells

**Fig. 8:**
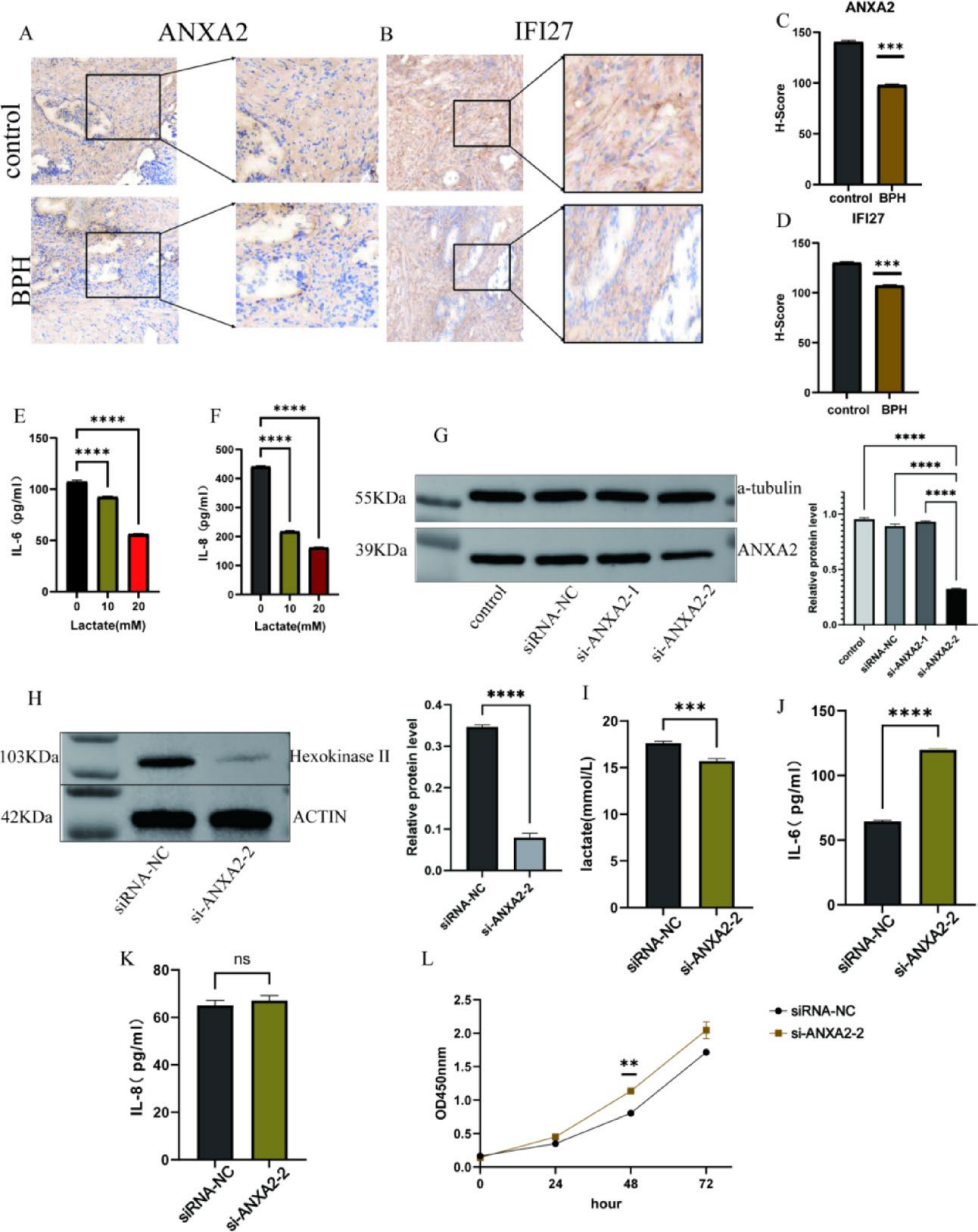
ANXA2 knockdown inhibits glycolysis and modulates the lactate-inflammation axis to promote BPH cell proliferation. **(A-B)** Immunohistochemistry images of normal prostate tissue and benign prostatic hyperplasia (BPH) tissue. Scale bar: 10 μm. **(C-D)** Quantitative analysis of ANXA2 and IFI27 expression levels using H-score. **(E-F)** ELISA assays measuring inflammatory cytokine secretion following treatment with graded concentrations of lactate. **(G)** Western blot and quantitative analysis for target sequence screening and validation of ANXA2 knockdown efficiency. Data are presented as mean ± SD from three independent biological replicates (*n* = 3). ****p < 0.0001. **(H)** Western blot and quantitative analysis of Hexokinase II protein expression following ANXA2 knockdown. Data are presented as mean ± SD from three independent biological replicates (*n* = 3). **(I)** Quantification of lactate levels using a lactate assay kit. **(J-K)** ELISA-based quantification of IL-6 and IL-8 secretion levels. **(L)** CCK-8 assay evaluating cell proliferation following ANXA2 knockdown.

IHC analysis suggests that the protein expression levels of ANXA2 and IFI27 in BPH tissues may be lower than those in normal prostate tissues, which is consistent with the trends observed in the transcriptomic analysis (Figs. 8A, C). Similarly, IFI27 showed higher staining intensity in normal prostate tissues, whereas its expression was relatively weaker in BPH (Figs. 8B, D). To explore the role of lactate in regulating inflammation in BPH cells, BPH cell lines were treated with varying concentrations of exogenous lactate. ELISA results demonstrated that as lactate concentration increased, the secretion levels of the pro-inflammatory cytokines IL-6 and IL-8 significantly decreased, suggesting that lactate may participate in the pathophysiological processes of BPH by modulating the inflammatory microenvironment (Figs. 8E-F). To identify the optimal target sequence and evaluate the functional role of ANXA2, we transfected cells with siRNAs and assessed knockdown efficiency using Western blot analysis. The results showed that ANXA2 expression was significantly reduced following siRNA transfection, with siRNA-ANXA2-2 achieving the highest knockdown efficiency (Fig. 8G). Further analysis revealed that silencing ANXA2 significantly reduced the expression of Hexokinase II, a key glycolytic enzyme (Fig. 8H), suggesting a potential impact on glycolysis-related metabolic processes. We next measured lactate concentrations and pro-inflammatory cytokine levels in the cell supernatant. Compared with the control group, the si-ANXA2 knockdown group exhibited a significant reduction in extracellular lactate (Fig. 8I). Meanwhile, IL-6 expression was markedly upregulated (Fig. 8J), whereas IL-8 levels remained unchanged (Fig. 8K). These findings suggest that reduced lactate levels following ANXA2 silencing may weaken lactate-mediated anti-inflammatory effects, resulting in the upregulation of specific inflammatory mediators such as IL-6. Finally, CCK-8 assays showed that ANXA2 knockdown significantly promoted the proliferation of BPH cells, with a marked increase in cell growth observed within 48 h (Fig. 8L). Taken together, these results indicate that although ANXA2 silencing suppresses glycolytic metabolism, the resulting alteration in the inflammatory microenvironment may paradoxically enhance BPH cell proliferation. Therefore, reduced ANXA2 expression may contribute to BPH development through dysregulation of the metabolic-inflammatory axis.

## Discussion

BPH is a highly prevalent condition among middle-aged and elderly men, characterized by a complex pathophysiological process involving inflammatory infiltration, hormonal imbalance, metabolic dysregulation, and extracellular matrix remodeling^23^. Protein lactylation, a recently identified post-translational modification tightly linked to cellular metabolic states, has been shown to play crucial regulatory roles in various diseases such as cancer and inflammation^24^. The analytical framework of this study draws upon the methodology previously established by our team^25,26^; however, its application here is novel. In this study, we innovatively integrated high-resolution insights from scRNA-seq with the robust analytical power of machine learning algorithms to systematically evaluate the expression status of lactylation-related genes across diverse cell types within BPH tissue at the single-cell level. Through this approach, we successfully identified core signature genes and critical cellular subpopulations closely associated with lactylation.

Remarkably, our findings revealed that ANXA2 and IFI27, two lactylation-related genes, were significantly downregulated in BPH tissues and exhibited highly cell-type-specific expression patterns-ANXA2 predominantly in epithelial cells and IFI27 mainly in endothelial cells. Moreover, their expression levels exhibited a strong negative correlation with the calculated cellular lactylation-related gene expression scores, suggesting that both genes may serve as potential indicators for reflecting or modulating the metabolic and lactylation status within BPH tissues. Further functional pathway and cell-cell communication analyses demonstrated that epithelial cell subpopulations with high ANXA2 expression were closely associated with inflammatory mediators, cell proliferation, and extracellular matrix interactions, whereas endothelial subpopulations with high IFI27 expression were linked to angiogenic signaling, androgen response, and immune cell chemotaxis. Collectively, these findings position ANXA2 and IFI27 as pivotal molecular nodes within the pathophysiological network of BPH, bridging key functional signaling pathways with potential lactylation-dependent regulatory mechanisms. This study thus provides novel molecular-level insights into the metabolic-inflammatory complexity underlying the pathogenesis of BPH.

ANXA2 is a multifunctional calcium-dependent phospholipid-binding protein that has been implicated in a variety of biological processes, including mediating Ca²⁺-regulated exocytosis and endocytosis, organizing lipid rafts, and regulating ion channels^27^. Its downregulation may compromise the membrane stability of prostatic epithelial cells, thereby disrupting intracellular and extracellular ion homeostasis and signal transduction, ultimately promoting abnormal hyperplasia. The gene expression of ANXA2 is also responsive to growth factors, further linking it to cell proliferation and transformation. In multiple cancer types, such as breast, gastric, and esophageal cancers^28–30^, ANXA2 is frequently reported to be upregulated and is believed to promote tumor invasion and metastasis by participating in membrane repair, cell migration, angiogenesis, and activation of signaling pathways such as EGFR and STAT3, often correlating with poor prognosis. However, previous studies have revealed significant heterogeneity in ANXA2 expression patterns in prostate cancer^31,32^. Tan et al. reported that ANXA2 expression was actually reduced in high-grade and advanced PCa cases and was associated with a higher risk of biochemical recurrence and metastasis^33^. Moreover, in vitro functional assays demonstrated that suppression of ANXA2 expression enhanced the invasive capacity of PC-3 cells^34^. These findings suggest that reduced ANXA2 expression in BPH may, under certain microenvironmental cues, serve as a precursor to malignant transformation. Low ANXA2 expression may lead to disrupted epithelial polarity and weakened cell-cell adhesion in BPH tissues, thereby promoting excessive epithelial proliferation and structural remodeling. Recent studies have revealed that ANXA2 interacts with the EGFR signaling pathway in tumor cells, which in turn activates glycolytic processes and leads to lactate accumulation^35^. Lactate primarily exerts suppressive and regulatory effects within inflammatory environments by directly modulating key signaling pathways and altering gene expression to restrain excessive inflammatory responses, thereby promoting the resolution of inflammation and the restoration of tissue homeostasis^36^. Chronic inflammation, particularly characterized by elevated IL-6, is a known clinical driver of BPH progression and is associated with larger prostate volumes and more severe lower urinary tract symptoms. We found that ANXA2 downregulation exacerbates IL-6 secretion, suggesting that the loss of ANXA2 may be a critical molecular trigger for “inflammation-driven” BPH. Therapeutic strategies targeting the ANXA2-lactate-inflammation axis may offer novel treatment avenues, especially for patients who respond poorly to conventional therapies.

IFI27 encodes an 11.5 kDa hydrophobic transmembrane protein^37^ that is primarily localized to the mitochondrial inner membrane, nuclear envelope, and endoplasmic reticulum^38^. This subcellular distribution suggests potential roles in cell signaling and energy metabolism. Mechanistic studies indicate that IFI27 exerts its biological functions through multiple pathways, including potential involvement in mitochondrial activity, apoptotic signaling, the promotion of immune cell infiltration, and participation in epigenetic modifications^39,40^. During infectious inflammation, IFI27 expression is markedly elevated, making it a critical biomarker of inflammatory responses^41^. As a downstream effector of inflammatory signaling, IFI27 is highly likely to participate in the pathogenesis and progression of BPH. In psoriasis, IFI27 is highly expressed in lesional tissues and contributes to keratinocyte proliferation^42^. Moreover, IFI27 has been linked to the glycolytic pathway in pancreatic cancer^43^, where glycolysis serves as the major source of pyruvate, which under specific conditions is converted into lactate. Downregulation of IFI27 may impair mitochondrial function, thereby preventing the metabolic shift toward glycolysis in BPH cells, reducing lactate production, and consequently suppressing protein lactylation, a process potentially involved in BPH pathogenesis. Collectively, these findings position IFI27 as a promising therapeutic target in BPH. Selective modulation of its expression or activity may help restore prostatic tissue homeostasis by influencing cellular energy metabolism, proliferation dynamics, and inflammation-driven glandular enlargement.

Our study reveals pronounced lactate metabolic features in BPH tissues, particularly within epithelial cells, a finding that is consistent with and extends previous research on prostate epithelial cell metabolism. Prior studies have demonstrated that normal prostate epithelial cells possess a distinctive energy metabolism pattern: unlike many other epithelial cell types, they preferentially perform incomplete glucose oxidation, converting a substantial proportion of glucose into lactate. Notably, TNF-α has been shown to enhance glycolysis and lactate production in prostate epithelial cells, driving metabolic reprogramming. Lactate, through mechanisms involving endoplasmic reticulum stress and apoptosis induction, modulates epithelial inflammatory responses; at physiological concentrations, it may exert anti-inflammatory effects, whereas pathological accumulation promotes inflammation^44^. Similarly, high-glucose environments directly induce metabolic shifts in prostate epithelial cells, characterized by increased glycolytic flux and altered lipid metabolism, resulting in elevated intracellular and microenvironmental lactate levels^45^. These metabolic changes further accelerate epithelial cell proliferation, reduce apoptosis, and enhance epithelial-mesenchymal interactions. The enhanced lactate metabolism observed in BPH epithelial cells in our study aligns with these findings, suggesting that glycolytic dysregulation and excess lactate accumulation may drive the characteristic epithelial hyperplasia and stromal remodeling in BPH.

Our results reveal a prominent lactylation-related gene expression signature in BPH tissues, specifically highlighting the significant enrichment of lactylation-related genes within the epithelial and endothelial cell populations. Lactylation-mediated metabolic reprogramming and gene regulation may play critical roles in epithelial cell proliferation and in endothelial cell-mediated support of angiogenesis and inflammation, representing a key component of BPH pathogenesis. Normal prostate epithelial cells exhibit a unique energy metabolism profile: unlike many other epithelial cell types, they preferentially perform incomplete glucose oxidation, converting a substantial portion of glucose into lactate. TNF-α has been shown to enhance glycolysis and lactate production in prostate epithelial cells. Previous studies have demonstrated that high-glucose environments directly affect prostate epithelial cells, inducing metabolic reprogramming characterized by increased glycolytic flux and altered lipid metabolism. This high-glucose-driven glycolytic enhancement elevates lactate levels both intracellularly and within the microenvironment, ultimately promoting accelerated epithelial proliferation, reduced apoptosis, and enhanced epithelial-mesenchymal transition.

Although this study provides novel insights, we acknowledge several limitations. First, the sample sizes of the training sets were relatively limited, and the control group in the validation set was notably small; this may affect the statistical reliability of the diagnostic performance evaluation for the biomarkers. Future systematic validation of the diagnostic value of ANXA2 and IFI27 in larger, multicenter prospective clinical cohorts is required. Second, while the BPH-1 cell line is widely utilized as an in vitro model for BPH due to its origin from patients with benign prostatic hyperplasia, it may not fully recapitulate the complete pathological characteristics of primary BPH tissue. Third, the absence of direct glucose uptake assays, extracellular acidification rate (ECAR) analysis, and standard rescue experiments renders the current mechanistic conclusions somewhat preliminary, necessitating further verification in subsequent studies.

## Conclusion

In summary, this study is the first to construct a comprehensive single-cell resolution atlas of lactylation-associated gene signatures in the BPH microenvironment. We successfully identified ANXA2 and IFI27 as key downregulated lactylation-related candidate genes in BPH. Our findings suggest that the loss of ANXA2 in prostate epithelial cells may influence a critical metabolic-inflammatory axis. These results provide important new insights into the complex interplay between metabolic reprogramming and chronic inflammation in BPH pathophysiology. Ultimately, this study highlights promising therapeutic targets for the future management of BPH.

## Supplementary Information

Below is the link to the electronic supplementary material.

Supplementary Material 1

Supplementary Material 2

Supplementary Material 3

Supplementary Material 4

Supplementary Material 5

Supplementary Material 6

Supplementary Material 7

Supplementary Material 8

Supplementary Material 9

Supplementary Material 10

Supplementary Material 11

Supplementary Material 12

Supplementary Material 13

Supplementary Material 14

## Data Availability

The datasets analyzed during the current study are publicly available. The bulk RNA-seq and scRNA-seq datasets were obtained from the NCBI Gene Expression Omnibus database (https://www.ncbi.nlm.nih.gov/geo/) under accession numbers: GSE132714 (https://www.ncbi.nlm.nih.gov/geo/query/acc.cgi?acc=GSE132714), GSE65343 (https://www.ncbi.nlm.nih.gov/geo/query/acc.cgi?acc=GSE65343), GSE104749 (https://www.ncbi.nlm.nih.gov/geo/query/acc.cgi?acc=GSE104749), GSE119195 (https://www.ncbi.nlm.nih.gov/geo/query/acc.cgi?acc=GSE119195), GSE7307 (https://www.ncbi.nlm.nih.gov/geo/query/acc.cgi?acc=GSE7307),GSE30994 (https://www.ncbi.nlm.nih.gov/geo/query/acc.cgi?acc=GSE30994),and GSE172357 (https://www.ncbi.nlm.nih.gov/geo/query/acc.cgi?acc=GSE172357).

## Code availability

The R code utilized for data analysis and figure generation is available on GitHub at https://github.com/good1000/lactylation1.
