# Supplementary material for: Integrating single-cell and bulk transcriptomic analyses to explore key lactylation-related genes in benign prostatic hyperplasia
Source: Sci Rep. 2026 May 25;16:23814. doi: 10.1038/s41598-026-54516-x (PMC13434622; doi:10.1038/s41598-026-54516-x)
Supplement: Supplementary file 1 [file 41598_2026_54516_MOESM14_ESM.docx]

**Supplementary Materials**

Supplementary Figure S1: Quality control and batch effect correction of single-cell RNA sequencing data. (A) Distribution of samples before and after batch correction. (B) UMAP density plots for cell type-specific marker genes.

Supplementary Figure S2. Principal component analysis (PCA) of the merged datasets before and after batch correction. (A) PCA plot before batch correction. Samples cluster distinctly according to their source datasets, indicating the presence of significant technical batch effects. (B) PCA plot of the merged datasets after batch correction. Samples from different datasets are harmoniously integrated and overlap with each other, demonstrating the successful elimination of cross-platform technical variations.

Supplementary Figure S3: Differential expression analysis of HMGA1 and NOP16. (A-B) Expression of HMGA1 and NOP16 in the training cohort. (C-D) Expression of HMGA1 and NOP16 in the validation cohort.

Supplementary Figure S4. Original full-length Western blot images corresponding to Figure 8G. The blots display the target sequence screening and validation of ANXA2 knockdown efficiency.

Supplementary Figure S5. Long-term validation of ANXA2 knockdown efficiency over a 120-hour experimental period. Western blot analysis compares the sustained protein expression levels of ANXA2 between the siRNA-NC and si-ANXA2-2 groups.

Supplementary Figure S6. Original full-length Western blot images corresponding to Figure 8H. The blots display the detection of Hexokinase II protein expression following ANXA2 knockdown.

Supplementary Tables S1-S6:

Table S1: Detailed list of 327 lactylation-related genes used in this study.

Table S2: List of 250 differentially expressed genes identified between high- and low-lactylation activity cell groups.

Table S3: List of 14 overlapping key lactylation-related genes identified in bulk RNA-seq analysis.

Table S4: Gene Ontology (GO) enrichment analysis results for the 14 overlapping key genes.

Table S5: Detailed signaling pathways associated with ANXA2⁺ epithelial cell communications.

Table S6: Detailed signaling pathways associated with IFI27⁺ endothelial cell communications.
